# Protocol for cerebrospinal fluid analysis using enrichment-enhanced surface-enhanced Raman spectroscopy and transformer-enabled spectral classification

**DOI:** 10.1016/j.xpro.2026.104538

**Published:** 2026-05-04

**Authors:** Zhaoyi Li, Dongjie Zhang, Zhaoyang Cheng, Hanju Zhang, Huandi Li, Lin Shi, Nan Wang, Qi Zeng, Xueli Chen

**Affiliations:** 1 Center for Biomedical-photonics and Molecular Imaging, Advanced Diagnostic-Therapy Technology and Equipment Key Laboratory of Higher Education Institutions in Shaanxi Province, School of Life Science and Technology, Xidian University, Xi’an, Shaanxi 710126, China; 2 Engineering Research Center of Molecular and Neuro Imaging, Ministry of Education & Xi’an Key Laboratory of Intelligent Sensing and Regulation of Trans-Scale Life Information, School of Life Science and Technology, Xidian University, Xi’an, Shaanxi 710126, China; 3 Bi-optoelectronic-integration and Medical Instrumentation Laboratory, Guangzhou Institute of Technology, Xidian University, Guangzhou, Guangdong 510555, China; 4 State Key Laboratory of Electromechanical Integrated Manufacturing of High-Performance Electronic Equipment, Xidian University, Xi’an, Shaanxi 710071, China

**Keywords:** Biophysics, Clinical Protocol, Chemistry, Material sciences, Computer sciences

## Abstract

The rapid detection and precise classification of cerebrospinal fluid in acute leukemia patients constitute a crucial clinical imperative. Here, we present a protocol for cerebrospinal fluid analysis deep learning with enrichment-enhanced surface-enhanced Raman spectroscopy (DL-SERS) detection and transformer-enabled spectral classification. We describe steps for preparing SERS-active materials and samples and acquiring SERS spectral data. We then detail procedures for deep learning-based classification. This protocol enables highly sensitive and efficient identification of acute leukemia patients.

For complete details on the use and execution of this protocol, please refer to Zhang et al.^1^

## Highlights

• Steps for preparing the surface-enhanced Raman scattering enhancement substrate
• Guidance on enrichment-assisted SERS detection of CSF
• Procedures for training transformer-enabled spectral classification model

## Abstract (editor highlights)

Publisher’s note: Undertaking any experimental protocol requires adherence to local institutional guidelines for laboratory safety and ethics.

## Graphical abstract

**Figure:**
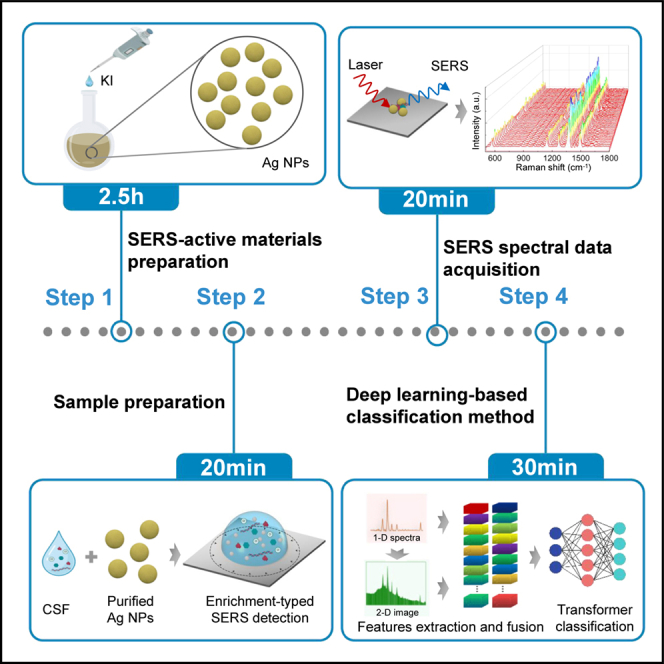

## Teaser

The rapid detection and precise classification of cerebrospinal fluid in acute leukemia patients constitute a crucial clinical imperative. Here, we present a protocol for cerebrospinal fluid analysis enrichment-enhanced surface-enhanced Raman spectroscopy (DL-SERS) detection and transformer-enabled spectral classification. We describe steps for preparing SERS-active materials and samples and acquiring SERS spectral data. We then detail procedures for deep learning-based classification. This protocol enables highly sensitive and efficient identification of acute leukemia patients.

## Before you begin

Central nervous system leukemia (CNSL) is a significant contributor to leukemia relapse and drug resistance, and may lead to severe complications or life-threatening conditions. Therefore, prompt identification of affected patients and timely administration of symptomatic treatment are critical.^2^^,^^3^ Currently, clinical diagnosis primarily relies on neurological symptom evaluation, cerebrospinal fluid (CSF) analysis, and neuroimaging studies. Among these methods, conventional cytology (CC) examination of CSF is considered the gold standard for confirming central nervous system involvement.^4^ However, its sensitivity is relatively low (<50%), often resulting in false-negative results, despite a specificity greater than 95%.^5^^,^^6^ Moreover, biomarkers released into the CSF by leukemic cells enable a diagnostic approach that is more sensitive, accurate, and quantifiable than traditional cytological techniques.^2^ These biomarkers can reflect key biological processes such as leukemic cell migration and adhesion to the central nervous system, metabolic plasticity, and intercellular interactions. As a result, biomarker-based detection and diagnosis of CNSL using CSF samples are increasingly becoming a focal point in current research.^7^

Surface-enhanced Raman scattering (SERS) is a molecular fingerprinting spectroscopic technique that offers high sensitivity, excellent specificity, rapid detection capability, suitability for in situ analysis, and the ability to simultaneously recognize multiple components, making it particularly advantageous in diverse analytical applications.^8^^,^^9^

The advancement of artificial intelligence (AI) has significantly accelerated the development and application of SERS technology. In particular, AI-integrated SERS strategy has been widely adopted for early cancer screening, disease subtyping and diagnosis, and assessment of therapeutic efficacy across various malignancies.^10^^,^^11^^,^^12^ In the field of leukemia research, Raman spectroscopy and SERS have attracted growing interest for their potential in detection and diagnosis. These techniques have demonstrated utility in differentiating acute myeloid leukemia (AML) cells from normal leukocytes, identifying specific chemical constituents and biomarkers, distinguishing among AML subtypes, and analyzing mutant gene expression profiles in leukemic cells.^13^^,^^14^ Despite these advancements, current AI-assisted SERS approaches predominantly rely on models such as convolutional neural networks or artificial neural networks, which typically classify samples based solely on 1D Raman spectra features, and this constrains the full exploitation of SERS data.^15^^,^^16^^,^^17^^,^^18^

### Innovation

Compared to existing techniques for leukemia detection, the proposed DL-SERS classification and diagnostic model demonstrates distinct methodological innovations and practical advantages. This protocol describes the steps for cerebrospinal fluid (CSF) analysis using Enrichment-Enhanced SERS detection and Transformer-Enabled spectral classification. In terms of sample analysis, the method enables rapid and convenient measurement, requiring only a minimal volume of cerebrospinal fluid to acquire spectral data within five min. For classification model, the model effectively integrates and concatenates 1D Raman spectral features with 2D image-based spectral characteristics, utilizing the fused feature set as input for subsequent training and testing. This multimodal strategy comprehensively captures multiple SERS spectral attributes, and substantially enhancing classification accuracy.

### Clean the experimental apparatus

**Timing: 2 h**

1. Immerse the flask and stir bar in aqua regia, and store in a dark place for 2 h.
2. Rinse the flask and stir bar with ultrapure water, followed by ultrasonic cleaning for 20 min. Repeat three times, and then dry the equipment in an oven.

***Note:*** Due to its volatility and strong corrosiveness, all operations with aqua regia must be conducted in a fume hood while wearing rubber gloves.

### Thaw CSF samples

**Timing: 0.5 h**

3. Passively thaw all CSF samples at room temperature (20°C–25°C) before any analysis.

***Note:*** Dry the outside of the cryovials thoroughly with clean lint-free paper to avoid contamination of the CSF samples.

### Hardware preparation

**Timing: 2 min**

4. Prepare the computer for the computational tasks.

***Note:*** All computational procedures required by this protocol can be completed using a standard personal computer, as its existing hardware setup is sufficient for the demands of data analysis and modeling. The system features a multi-core CPU that delivers robust processing performance, complemented by a GPU that supports machine learning–related computations. In addition, the machine is configured with ample memory capacity to ensure efficient handling of large datasets. Storage resources are also adequate for accommodating the substantial volumes of data produced during the study, while external storage devices are utilized to support data backup and long-term archiving.

***Note:*** The experiments were conducted on a desktop workstation configured with an Intel® Core™ i7-10700K CPU, an NVIDIA GeForce RTX 3090 GPU, 32 GB of DDR4 RAM, and a 1 TB solid-state drive, operating under the Windows 11 system. This combination of hardware components provides stable and efficient performance for handling high-intensity computational workloads. It should be noted that while the experimental work in this study was conducted on this specific configuration, the proposed methodology remains compatible with different processor and graphics card combinations and can be flexibly adapted to various computational environments.

### Software preparation

**Timing: 1 h**

The computational implementation of this methodology requires the prior establishment of a corresponding software foundation. This prerequisite entails the complete installation and configuration of Python and Anaconda within the operating system, alongside the integration of key software libraries including, but not limited to, Numpy, Matplotlib, Pandas and Seaborn. As essential functional modules, all specified packages must be properly configured to ensure the successful execution and reproducibility of the subsequent research procedures.

5. Install software.
  a. Install Anaconda.
  b. Install PyTorch.
  c. Create a Python environment.

***Note:*** The adoption of a virtual environment facilitates the isolation and independent management of the dependencies required for this protocol, thereby minimizing the risk of dependency conflicts with other projects.

6. Install required packages.
  a. Install Numpy: Numpy provides efficient multidimensional array objects and a comprehensive set of array manipulation tools.
    >pip install numpy
  b. Install Matplotlib: Matplotlib provides a comprehensive 2D plotting capability for the efficient creation of high-quality, diverse visualizations.
    >pip install matplotlib
  c. Install Pandas: Pandas provides efficient and expressive data structures for intuitive manipulation and analysis of labeled data.
    >pip install pandas
  d. Install Seaborn: Seaborn provides a high-level statistical visualization interface for the straightforward creation of complex, aesthetically pleasing visualizations.
    >pip install seaborn

## Key resources table

**Table:** 

REAGENT or RESOURCE	SOURCE	IDENTIFIER
**Biological samples**		

cerebrospinal fluid	This paper	N∖A

**Chemicals, peptides, and recombinant proteins**		

Silver nitrate (0.1000 mol/L)	Aladdin	S116267; CAS No.: 7761-88-8(solution)
Sodium citrate (≥99%)	Aladdin	S189183; CAS No.:68-04-2
Potassium iodide (≥99.0%)	Sigma	CAS No.: 7681-11-0
Perfluoropolyether lubricating oil (GPL 107)	Dupont Krytox	CAS No.: 60164-51-4

**Critical commercial assays**		

Ultraviolet-visible spectroscopy	Agilent	Carry 60
Confocal Raman Spectrometer	Renishaw	Invia system

**Software and algorithms**		

Python (version 3.8.16)	Python Software Foundation	https://www.python.org
PyTorch (version 2.1.1+cu121)	Python Software Foundation	https://www.python.org
Numpy (version 1.23.5)	Python Software Foundation	https://www.python.org
Matplotlib (version 3.7.1)	Python Software Foundation	https://www.python.org
Pandas (version 2.0.3)	Python Software Foundation	https://www.python.org
Seaborn (version 0.12.2)	Python Software Foundation	https://www.python.org
Cuda (version 12.2)	NVIDIA Developer	https://developer.nvidia.com
Cuda (version 8.8.1)	NVIDIA Developer	https://developer.nvidia.com
Matlab (version 2023a)	MathWorks Developer	https://www.mathworks.com/

## Materials and equipment

**Table:** 

Reagent	Final concentration	Amount
silver nitrate	1mM	1 mL in 99 mL
sodium citrate	1%wt	0.1 g in 10 mL
potassium iodide	1mM	1.66 mg in 10 mL
**Total**	**N/A**	**120 mL**

***Note:*** The solution can be stored at 4°C for one month.

## Step-by-step method details

### Synthesis of AgNPs

**Timing: 2 h (for step 1 to step 6)**

**Timing: 0.5 h (for step 7 to step 9)**

***Note:*** Prior to initiating the subsequent experimental procedures, the operator is required to don a laboratory coat, protective gloves, and a face mask to avoid direct contact between the skin and solid or liquid chemical substances, which could otherwise result in physical irritation or discomfort.

This section aims to prepare silver nanoparticles (Ag NPs) using the classic sodium citrate reduction method and subsequently purify them, so that they can be used for subsequent surface-enhanced Raman scattering (SERS) spectroscopy measurements.

Ag nanoparticles were prepared by the classical sodium citrate reduction method. To mitigate interference from surface adsorbates on the measured surface-enhanced Raman scattering (SERS) spectra, the silver nanoparticles were purified using a potassium iodide solution.^19^

1. The preparation of All Solutions Required.
  a. Weigh out 0.1 g of solid sodium citrate using an electronic balance and dissolve it in 10 mL of ultrapure water.
  b. Weigh out 1.66 mg of solid potassium iodide using an electronic balance and dissolve it in 10 mL of ultrapure water.
2. Measure out 100 mL of a 1 mM silver nitrate solution and transfer it into a dry 200mL round-bottom flask equipped with a magnetic stir bar.
3. Heat the conical flask at 110°C until the solution reaches boiling.
4. Add 1 mL of 1%wt sodium citrate solution to the boiling solution.
5. Continue stirring the reaction solution for 1 h until its color turns yellow-green.
6. Store the obtained yellow-green Ag NPs solution at 4°C.

**CRITICAL:** After use, the Ag NPs should be kept in a refrigerator at 4°C in the dark environment, with a maximum allowable storage period of three months.

***Note:*** To ensure long-term stability, the storage container must remain hermetically sealed to prevent moisture ingress, which could otherwise compromise sample integrity and analytical reproducibility.

**Pause point:** Prior to mixing with CSF, Ag NPs were aged at 4°C for one week.

7. Mix the silver nanoparticle solution with 1 mM KI solution at a 1:1 volume ratio.
8. Allow the mixture to react at room temperature for 20 min.
9. Centrifuge the resulting solution, discard the supernatant, and concentrate it to five times its original concentration.

**CRITICAL:** The mixture of Ag NPs and KI must be used immediately after preparation and should not be stored.

***Note:*** Iodide ions (I^-^) selectively adsorb onto the Ag surface to form strong Ag-I bonds, displacing citrate and preventing its re-adsorption. This process fully suppresses citrate-derived spectral interference.

### Enrichment-based SERS measurement

**Timing: 20 min (for step 10 to step 11)**

**Timing: 20 min (for step 12)**

This section aims to perform SERS spectroscopy measurements and data acquisition on cerebrospinal fluid (CSF) samples using an enrichment-type substrate combined with the surface-enhanced effect of Ag NPs.

10. SERS Substrate Preparation.
  a. Fix a piece of Teflon membrane onto a 5 cm × 5 cm flat glass slide using double-sided adhesive tape.
  b. Apply 0.5 mL of DuPont GPL105 perfluorinated lubricant onto the Teflon membrane surface and distribute it uniformly via spin coating.
  c. Bake the coated substrate at 80°C for 10 min. The resulting substrate is ready for use as an enhanced SERS substrate.

**CRITICAL:** Ensure the Teflon membrane remains clean and lies perfectly flat on the glass slide.

***Note:*** In the physical enrichment process, a lubricant-infused porous surface (SLIPS) method was employed, utilizing a PTFE microporous membrane infused with perfluoropolyether oil to prepare a hydrophobic substrate. This approach significantly mitigates the coffee ring effect on the substrate surface, thereby facilitating droplet sliding and condensation, which concentrates the droplet into a smaller central region. The dense distribution of nanoparticles effectively reduces interparticle spacing, thereby substantially enhancing SERS hotspots and improving detection sensitivity.

11. Sample and Silver Nanoparticle Mixing.
  a. Mix CSF specimens with the silver nanoparticle solution at a 1:1 volume ratio.
  b. Gently pipette the mixture to ensure thorough homogenization.

***Note:*** The CSF samples obtained from the hospital were not subjected to any additional processing other than thawing.

12. SERS Detection Substrate Preparation and Data Acquisition.
  a. Aspirate 1–2 μL of the mixture using a pipette and droplet-cast it onto the Teflon-based substrate.
  b. Place the substrate with the deposited sample at 80°C to evaporate the solvent, forming the final SERS detection substrate within 2 min.
  c. Obtain Raman spectra with a confocal Raman spectrometer set to a 532 nm excitation wavelength, a grating density of 1800 lines/mm, a spectral resolution of 0.95 cm^−1^, a spectral range of 100–3000 cm^−1^, a 50× objective (N.A.=0.5), 0.35 mW laser power, an integration time of 1 second, and 1 accumulation.
  d. For each CSF sample, gather a minimum of 50 valid Raman spectra from systematic grid-based sampling in dry sample areas.

***Note:*** The laser power is measured at the sample surface.

### Deep learning-based classification method

**Timing: 0.5 h**

This section aims to classify the preprocessed SERS spectral data using deep learning methods, in order to achieve differential diagnosis of leukemia and its subtypes in cerebrospinal fluid (CSF) samples.

13. Spectral data preprocessing.
  a. Process raw Raman spectra using Savitzky-Golay filtering for smoothing and noise reduction.
    >y_filter = savgol_filter(y, 21, 5, mode="nearest")
  b. Subtract background signals by applying morphology-based iterative baseline correction to the smoothed spectra.
    >y_baseline_correction=y_uniform-y_baseline
  c. Convert the preprocessed 1D spectral data into 2D image representations.
    ***Note:*** Define Raman shift as the independent variable (X-axis) and the corresponding intensity as the dependent variable (Y-axis), ensuring a strict one-to-one mapping for all data points.
  d. Resample spectral data points using linear interpolation, minimizing the residual sum of squares between adjacent points.
  e. Generate a continuous spectral curve by sequentially connecting all interpolated data points with a solid line.
  f. Interpret the final 2D image as a spatial distribution map of Raman intensity across the defined shift range.
    ***Note:*** The variations in spectral peak profiles visually represent the signal distribution.
  g. Input the converted 2D image data into a convolutional neural network for feature extraction and integrate it within the classification framework for multi-modal fusion.
    >set(gcf,'Position',[0 ,0 ,328 ,328])
    >picName =['AL-',num2str(i+4284),'.png']
14. Feature extraction and fusion.
  a. Apply Principal Component Analysis (PCA) to the preprocessed 1D SERS spectral data for dimensionality reduction and feature extraction.
  b. Set the number of principal components to 1024, ensuring the cumulative variance contribution rate of the selected components exceeds 95%.
    >pca = PCA(n_components=1024)
    >pca.fit(y_uniform)
    >low_d = pca.transform(y_uniform)
    >low_d = low_d.reshape((2888, 1, 1024)).astype('float64')
    >variances = pca.explained_variance_ratio_ ∗ 100
    >cumulative_variances = np.cumsum(variances)
  c. Use these 1024 principal components as 1D input features for subsequent analysis.
  d. Utilize a ResNet50 deep convolutional neural network to extract features from the generated 2D SERS spectral images.
  e. Crop all input 2D spectral images to a central 224 × 224 pixel region to meet ResNet50’s input requirements.
  f. Feed the cropped images into the pre-trained ResNet50 model and extract the resulting high-level features.
  g. Combine the 1D features obtained from PCA with the 2D image features extracted by ResNet50.
  h. Concatenate these two feature vectors via matrix stacking to form an integrated feature set.
  i. Employ this fused feature set for the final SERS spectral classification.
    >e=np.concatenate((low_d, data_npy),axis=2)
    >classdata = np.concatenate((e,label),axis=2)
15. Transformer classification method.
  ***Note:*** The Transformer-based classification model constructed in this study primarily consists of a linear embedding layer, a multi-head self-attention mechanism (with 4 attention heads), an adaptive average pooling layer, and a multi-layer perceptron.
  a. Define the classification tasks by specifying six distinct categories for the model, including but not limited to: normal vs. leukemia (AL), AML vs. ALL, and B-ALL vs. T-ALL.
  b. Assemble the model components.
    ***Note:*** Design a Transformer architecture that sequentially consists of an embedding layer, a multi-head attention layer, a multilayer perceptron (MLP) comprising two linear layers and a Dropout layer, and concludes with a fully connected output layer.
  c. Feed the fused 1D and 2D features into the embedding layer.
    >self.embedding = nn.Linear(input_dim, hidden_dim)
  d. Generate Q, K, V vectors by projecting the embedded vectors through separate linear transformations into query (Q), key (K), and value (V) vectors.
  e. Compute the attention weights by multiplying the Q and K matrices, and then normalize the resulting values.
  f. Apply the attention weights to perform a weighted sum of the V vectors, and add a Dropout layer with a rate of 0.1.
    ***Note:*** This step is designed to apply the attention mechanism and mitigate overfitting.
    >scores = torch.matmul(q, k.permute(0, 1, 3, 2))
    >attn_weights = torch.softmax(scores, dim=-1)
    >attended_vals = torch.matmul(attn_weights, v)
    >attended_vals = self.norm1(attended_vals)
    >attended_vals = self.dropout(attended_vals)
  g. Produce the attention output.
    i. Normalize and linearly transform the weighted sum.
    ii. Combine the result with the original input vector.
      >output = self.fco(attended_vals)
  h. Perform adaptive average pooling on the attention output.
    ***Note:*** This step is performed to reduce feature dimensionality, compress data, and extract key features.
    > self.pooling = nn.AdaptiveAvgPool1d(1)
  i. Process data through the MLP.
    i. Input the fused features into the module, using ReLU as the activation function between the two linear layers.
    ii. Apply a Dropout rate of 0.1 after each linear layer.
  j. Transform the data through the fully connected layer to the required output dimension to generate the classification output.
    i. For binary classification, apply the Sigmoid activation function to convert outputs into probabilities between 0 and 1.
    ii. For multi-class tasks, use the Softmax function to generate probability distributions across all classes.
      >self.fc = nn.Linear(hidden_dim, output_dim)
      >self.sigmoid = nn.Sigmoid()
      ***Note:*** The dataset was split into training, validation, and test sets in an 8:1:1 ratio.
  k. Select BCELoss for binary classification and CrossEntropyLoss for multi-class classification to configure the loss function and optimizer.
    ***Note:*** For all tasks, employ the Adam optimizer with a unified learning rate of 0.0001 and a batch size of 2 to update model parameters.
    >criterion = nn.BCELoss()
    >optimizer = optim.Adam(model.parameters(), lr=0.0001)
  l. Select the optimal hyperparameters using a dynamic model selection strategy based on the validation set.
    ***Note:*** During the 300-epoch training process, validation accuracy was monitored in real time, and only the weight model with the best validation performance was saved and used for final testing.

## Expected outcomes

### Characterization of SERS-enhanced substrates

**Figure 1:**
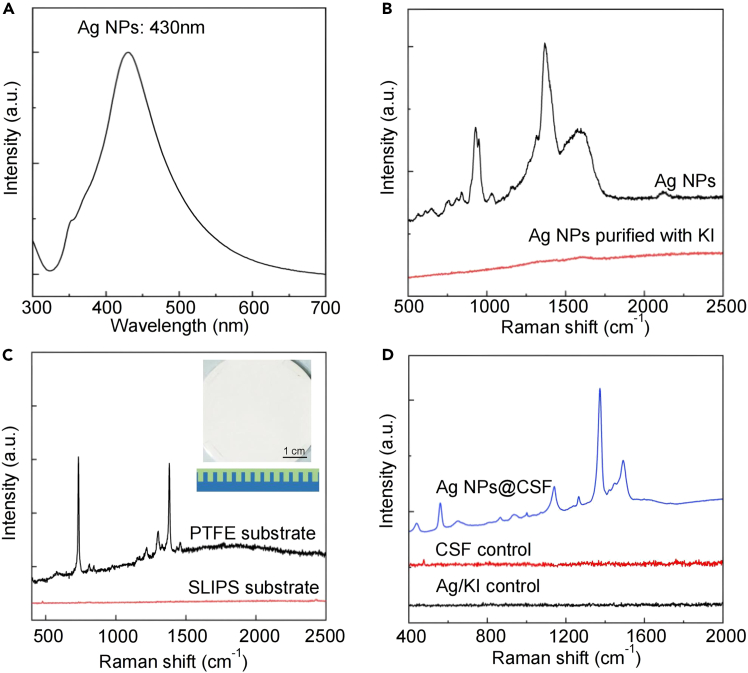
Characterizations of SERS enhanced substrates (A) UV-vis absorption spectra of Ag NPs. (B) SERS spectra of Ag NPs before and after purified with KI agent. (C) SERS spectra of PTFE membrane and slippery liquid-infused porous surface (SLIPS) substrate. The insets show the optical image of SLIPS substrate. (D) SERS spectra of Ag/KI NPs control, CSF control (from AML patient), and mixture of Ag NPs and CSF. Reproduced from Zhang et al.^1^

Silver nanoparticles (Ag NPs) colloids exhibiting a surface plasmon resonance peak at 430 nm were selected as SERS-active substrates. Prior to Raman signal acquisition, the Ag NP colloids underwent a series of pretreatment steps, including concentration, purification, and thorough mixing (Figure 1A). For surface cleaning, a 1 mM KI solution was introduced, which effectively removed background signals and potential interferents, yielding clean and interference-free Raman spectra (Figure 1B).

Slippery liquid-infused porous surfaces (SLIPS) were employed as the supporting substrates to facilitate the evaporation and deposition of cerebrospinal fluid (CSF) and Ag NP mixtures. Raman measurements confirmed that the pristine SLIPS substrate itself produced negligible background signals (Figure 1C). When CSF was combined with Ag NPs on the SLIPS substrate, a SERS spectrum with a high signal-to-noise ratio was obtained. In contrast, neither the CSF sample nor the Ag NPs treated with KI alone generated discernible Raman features. Through the dual strategies of physical enrichment and chemical purification, it effectively ensured that no interfering signals were introduced by each material during the sample preparation process, thereby guaranteeing the accuracy and reliability of the spectral detection (Figure 1D).

**Figure 2:**
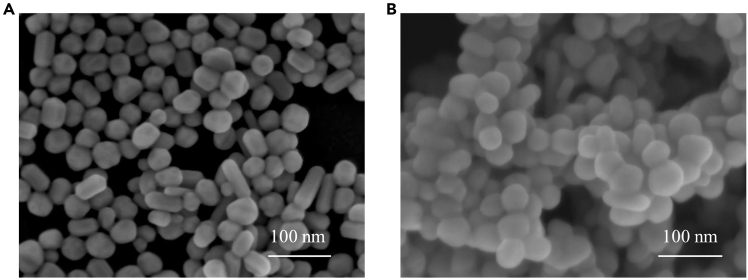
SEM images of colloidal Ag NPs with different aggregation station (A) Ag NPs purified with KI. (B) Ag NPs mixed with CSF sample. Reproduced from Zhang et al.^1^

The morphological characteristics of Ag NPs before and after interaction with CSF were examined using scanning electron microscopy (SEM), as presented in Figure 2.

### SERS performance of the synthesized substrates

**Figure 3:**
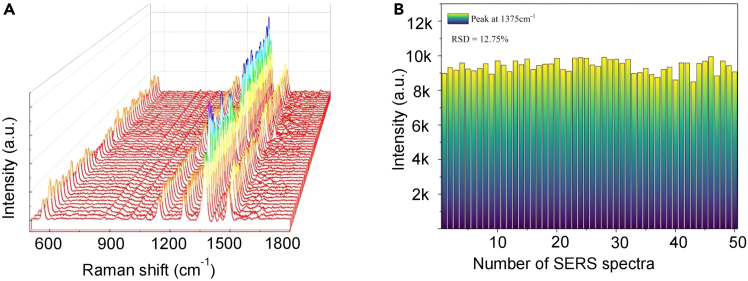
The performance of SERS uniformity (A) 50 of SERS spectra collected from one sample. (B) The relative standard deviation (RSD) value of characteristic band at 1375 cm^−1^.

The images distinctly showed significant nanoparticle aggregation after interaction with CSF. The reproducibility of the SERS measurements was subsequently evaluated (Figure 3), indicating high spectral consistency, with a relative standard deviation (RSD) of 12.75% observed at the characteristic Raman peak at 1,375 cm^−1^.

### Spectroscopic classification results

**Figure 4:**
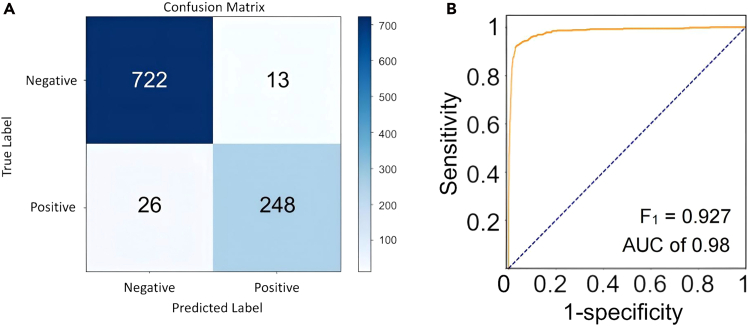
Classification of acute leukemia and healthy group (A) The confusion matrix of binary classification for screening of acute leukemia and healthy individuals. (B) The ROCs of binary classification for screening of acute leukemia and healthy individuals.

Due to the considerable inter-individual variability present in CSF samples, relying solely on characteristic peak positions or intensities proved insufficient for accurate differentiation. Consequently, direct discrimination between healthy and diseased individuals based on these features alone was not feasible. The assembled dataset, which encompasses spectral features and their variations in patient samples, was subsequently employed for model training, validation, and testing. A deep learning framework based on a transformer architecture combined with multimodal feature fusion of one-dimensional spectral data and two-dimensional spectral images was implemented. This approach effectively captures key variations in spectral features, such as differences in intensity and shifts in peak positions across critical wavenumber regions. The resulting confusion matrix demonstrated a classification accuracy of 96.13% in differentiating healthy subjects from leukemia patients (Figure 4A). Moreover, the model achieved an F1 score of 0.927 and an area under the ROC curve (AUC) of 0.98 (Figure 4B), indicating excellent diagnostic sensitivity and specificity. The results indicate that SERS spectroscopy combined with multivariate analysis methods holds promising diagnostic potential for the detection of acute leukemia in cerebrospinal fluid.

## Limitations

The enrichment-based SERS enhancement strategy proposed in this study has so far demonstrated efficacy exclusively in cerebrospinal fluid specimens, enabling detection at trace levels with high sensitivity. For other biological fluids, such as, blood, urine, or tear samples, further methodological optimization is required to achieve comparable analytical performance.

## Troubleshooting

All reagents and materials employed in this protocol may be replaced with equivalent products from alternative suppliers. However, it is recommended to use the reagents listed in the ‘key resources table’ to maintain experimental reproducibility.

### Problem 1

During silver nanoparticle synthesis, high-temperature boiling causes continuous water evaporation. This steadily increases reactant concentrations, deviating from the intended stoichiometric ratio. Given the high concentration sensitivity of nanoparticle nucleation and growth, such deviations cause heterogeneous particle sizes and irregular morphologies, ultimately compromising experimental reproducibility.

### Potential solution

During heating and boiling, attach a reflux condenser to the reaction vessel. Circulate cooling water through the condenser to ensure that evaporated solvent vapor condenses on the inner wall and returns to the reaction system.

### Problem 2

When left unused for extended periods, prepared SLIPS may experience degraded hydrophobic surface properties and diminished enrichment performance.

### Potential solution

The prepared SLIPS substrate should be used promptly after fabrication to maintain optimal performance.

### Problem 3

Conventional excitation wavelengths (e.g., 633 nm, 785 nm) yield suboptimal SERS signals from CSF. Excessive laser power induces sample damage, resulting in unreliable data acquisition.

### Potential solution

Raman spectra of CSF were acquired following the data collection procedure described in Step 12.

### Problem 4

The presence of anomalous data degrades the performance of classification models.

### Potential solution

Manually exclude SERS spectra exhibiting cosmic ray interference or signal saturation during spectral acquisition.

### Problem 5

The loss further diminishes during the convergence phase of training.

### Potential solution

During the model training convergence phase, the persistent reduction in training loss accompanied by plateaued or degraded validation performance unequivocally manifests a quintessential hallmark of overfitting. This phenomenon suggests that the model is predominantly assimilating specific sample attributes and extraneous noise from the training dataset, rather than acquiring the fundamental data patterns with robust generalization capabilities. It is imperative to implement a termination criterion predicated on validation loss metrics and suspend the training process when no discernible enhancement is detected across successive epochs. The model was trained for a fixed period of 300 epochs, effectively avoiding fluctuations caused by overfitting in the later stages of training.

## Resource availability

### Lead contact

Further information and requests for resources should be directed to and will be fulfilled by the lead contact, Xueli Chen (xlchen@xidian.edu.cn).

### Technical contact

Technical questions on executing this protocol should be directed to and will be answered by the technical contact, Dongjie Zhang (zhangdongjie@xidian.edu.cn).

### Materials availability

This study did not generate new unique reagents.

### Data and code availability

All data and any additional information are available from the lead contact upon request.

• The code and data, including the model, the training engine, the testing script, model weights, and partial test dataset, have been deposited at Zenodo and are publicly available as of the date of publication.
• Any additional information required to reanalyze the data reported in this paper is available from the lead contact upon request.

## Acknowledgments

This work was supported by the 10.13039/100016698National Natural Science Foundation of China, grants 32201133 and 62275210; the National Leading Talent Program; the National Young Talent Program; the Shaanxi Young Top-notch Talent Program; the Young Talent Fund of the Shaanxi Young Top-notch Talent Program in Shaanxi, grant 20230222; the 10.13039/501100021171Guangdong Basic and Applied Basic Research Foundation, grant 2025A1515010789; the 10.13039/501100012226Fundamental Research Funds for the Central Universities, grants ZYTS25098 and QTZX25113; and the Opening Foundation of Shanxi Key Laboratory of Ferroelectric Physical Micro-nano Devices and Systems, grant TDWL202501. Raman measurement and sample preparation were supported by the Instrument Analysis Center of Xidian University and the Comprehensive Experimental Center for Chemistry and Bioscience of Xidian University.

## Author contributions

D.Z. and X.C. conceived and designed the study. Z.L. and H.L. conducted the experiments and characterizations. Z.C. and N.W. implemented the deep learning algorithm. Z.L., H.Z., Z.C., and L.S. provided support for data analysis. X.C., D.Z., and Q.Z. supervised the study. D.Z., Z.L., and X.C. wrote and revised the manuscript.

## Declaration of interests

The authors declare no competing interests.

## References

[1] Zhang D., Cheng Z., Song Y., Li H., Shi L., Wang N., Peng Y., Chen R., Sun N., Han M. (2025). Rapid and sensitive acute leukemia classification and diagnosis platform using deep learning-assisted SERS detection. Cell Rep. Med..

[2] Paul S., Short N.J. (2022). Central Nervous System Involvement in Adults with Acute Leukemia: Diagnosis, Prevention, and Management. Curr. Oncol. Rep..

[3] Thastrup M., Duguid A., Mirian C., Schmiegelow K., Halsey C. (2022). Central nervous system involvement in childhood acute lympho-blastic leukemia: challenges and solutions. Leukemia.

[4] Del Principe M.I., Buzzatti E., Piciocchi A., Forghieri F., Bonifacio M., Lessi F., Imbergamo S., Orciuolo E., Rossi G., Fracchiolla N. (2021). Clinical significance of occult central nervous system disease in adult acute lymphoblastic leukemia. A multicenter report from the Campus ALL Network. Haematologica.

[5] Co'rdova-Serrano R.D., Almanza Huante E., Ma'rtinez-Moreno E., He'r-nandez-Alca'ntara A., Ferna'ndez-Sa'nchez E., Gomez-De Leon A., Espinosa K.A. (2020). Central Nervous System (CNS) Involvement in Adult Acute Lymphoblastic Leukemia (ALL) Assessed Solely By Flow Cytometry Has an Adverse Impact on Survival. Blood.

[6] Kopmar N.E., Cassaday R.D. (2023). How I prevent and treat central nervous system disease in adults with acute lymphoblastic leukemia. Blood.

[7] Lenk L., Alsadeq A., Schewe D.M. (2020). Involvement of the central nervous system in acute lymphoblastic leukemia: opinions on molecular mechanisms and clinical implications based on recent data. Cancer Metastasis Rev..

[8] Cutshaw G., Uthaman S., Hassan N., Kothadiya S., Wen X., Bardhan R. (2023). The Emerging Role of Raman Spectroscopy as an Omics Approach for Metabolic Profiling and Biomarker Detection toward Precision Medicine. Chem. Rev..

[9] Liu J., Zheng T., Tian Y. (2019). Functionalized h-BN Nanosheets as a Theranostic Platform for SERS Real-Time Monitoring of MicroRNA and Photodynamic Therapy. Angew. Chem. Int. Ed. Engl..

[10] Dong S., He D., Zhang Q., Huang C., Hu Z., Zhang C., Nie L., Wang K., Luo W., Yu J. (2023). Early cancer detection by serum biomolecular fingerprinting spectroscopy with machine learning. eLight.

[11] Shi L., Li Y., Li Z. (2023). Early cancer detection by SERS spectroscopy and machine learning. Light Sci. Appl..

[12] Yi J., You E.M., Liu G.K., Tian Z.Q. (2024). AI–nano-driven surface-enhanced Raman spectroscopy for marketable technologies. Nat. Nanotechnol..

[13] Ayyappan V., Chang A., Zhang C., Paidi S.K., Bordett R., Liang T., Barman I., Pandey R. (2020). Identification and Staging of B-Cell Acute Lymphoblastic Leukemia Using Quantitative Phase Imaging and Machine Learning. ACS Sens..

[14] Oktem F., Akdeniz M., Al-Shaebi Z., Akyol G., Keklik M., Aydin O. (2025). SERS and machine learning-enabled liquid biopsy: a promising tool for early detection and recurrence prediction in acute leukemia. ACS Omega.

[15] Shin H., Choi B.H., Shim O., Kim J., Park Y., Cho S.K., Kim H.K., Choi Y. (2023). Single test-based diagnosis of multiple cancer types using Exosome-SERS-AI for early stage cancers. Nat. Commun..

[16] Huang L., Sun H., Sun L., Shi K., Chen Y., Ren X., Ge Y., Jiang D., Liu X., Knoll W. (2023). Rapid, label-free histopathological diagnosis of liver cancer based on Raman spectroscopy and deep learning. Nat. Commun..

[17] Xie Y., Su X., Wen Y., Zheng C., Li M. (2022). Artificial Intelligent Label-Free SERS Profiling of Serum Exosomes for Breast Cancer Diagnosis and Postoperative Assessment. Nano Lett..

[18] Shin H., Oh S., Hong S., Kang M., Kang D., Ji Y.G., Choi B.H., Kang K.W., Jeong H., Park Y. (2020). Early-Stage Lung Cancer Diagnosis by Deep Learning-Based Spectroscopic Analysis of Circulating Exosomes. ACS Nano.

[19] Xu L.J., Zong C., Zheng X.S., Hu P., Feng J.M., Ren B. (2014). Label-free detection of native proteins by surface-enhanced Raman spectroscopy using iodide-modified nanoparticles. Anal. Chem..

